# Bioenergetics of the *Dictyostelium* Kinesin-8 Motor Isoform

**DOI:** 10.3390/biom10040563

**Published:** 2020-04-07

**Authors:** Michael P. Koonce, Irina Tikhonenko

**Affiliations:** Division of Translational Medicine, Wadsworth Center, NYS Department of Health, Albany, NY 12237, USA; irina.tikhonenko@health.ny.gov

**Keywords:** kinesin, microtubules, *Dictyostelium*, cell motility

## Abstract

The functional organization of microtubules in eukaryotic cells requires a combination of their inherent dynamic properties, interactions with motor machineries, and interactions with accessory proteins to affect growth, shrinkage, stability, and architecture. In most organisms, the Kinesin-8 family of motors play an integral role in these organizations, well known for their mitotic activities in microtubule (MT) length control and kinetochore interactions. In *Dictyostelium discoideum*, the function of Kinesin-8 remains elusive. We present here some biochemical properties and localization data that indicate that this motor (DdKif10) shares some motility properties with other Kinesin-8s but also illustrates differences in microtubule localization and depolymerase action that highlight functional diversity.

## 1. Introduction

The Kinesin-8 family of proteins are microtubule (MT)-based molecular motors that largely function in polymer length control and are required for chromosome alignment, mitotic spindle length determination, and astral MT dynamics in a number of animal and fungal cells [1,2]. Though primarily studied for their mitotic activities, these motors are also known to function during interphase, to facilitate cell polarity, nuclear position, and MT organization [3,4,5,6]. We previously addressed Kinesin-8 function in *Dictyostelium discoideum* through a gene knockout of the Dd*Kif10* isoform, and were unable to detect any significant phenotypic consequences of its removal [7]. Null cells grow and develop in a manner indistinguishable from wild type cells, and there are no apparent alterations in the interphase or mitotic MT patterns. The only substantial alteration we could find was an inability to produce the distinctive comet-like MT array movement in DdKif10 null cells, an effect that results from the dominant negative overexpression of the dynein motor domain. This same result is also seen in Kinesin-4 (Dd*Kif8*) knockout cells, but not in other kinesin nulls, suggesting that both DdKif8 and DdKif10 collaborate with dynein to facilitate the organization of the radial interphase MT array [7].

This brief report presents some of the biochemical properties of the DdKif10 motor. Previous works in other organisms indicate that Kinesin-8 isoforms are relatively slow, plus-end directed motors, some with distinctive MT depolymerization activities [2]. We show, here, in vitro gliding and ATPase activities, as well as the in vivo localization of a motor fragment. Moreover, we test the idea that DdKif8 and DdKif10 cooperate their activities in interphase cells.

## 2. Materials and Methods

### 2.1. Molecular Constructs

The full-length coding sequence for Dd*Kif10* (3714bp, 1238aa), was obtained from the genome resources at DictyBase (DDB_G0293198) [8]. The cDNA sequence, minus the stop codon was commercially synthesized (GenScript, Piscataway, NJ, USA) and subcloned into an expression vector containing the DdActin15 promotor, an amino terminal 8x His affinity tag, and a G418 selectable marker [9]. We added the 732bp GFP (S65T) sequence onto the carboxy-terminus of the full-length version and also prepared two shorter variants using PCR, a mid-length version presented here (725aa), and a motor/neck domain-only fragment (439aa). All three constructs were verified by sequencing and introduced into *D. discoideum* AX-2 cells for expression [10]. Only the mid-length fragment produced detectable levels of polypeptide.

### 2.2. Biochemistry and Imaging

Protein from these cells was affinity purified following the methods in [9]. Twice-cycled tubulin was extracted from fresh beef brain, and further purified using phosphocellulose chromatography. MT binding assays were performed in BRB80 buffer (80 mM PIPES, pH 6.8, 1 mM MgCl_2_, 1 mM EGTA), 6 μM of motor was added to paclitaxel-stabilized MTs (0–25 μM) in 200 μL reactions, allowed to bind 15 min (20 °C), and sedimented at 150,000g (15 min). Supernatants were removed, pellets were resuspended in 200 μL BRB80, and aliquots were run on 7.5% SDS-gels and Coomassie stained. Band intensities were quantitated, and curve fittings were performed using FIJI [11]. ATPase assays were performed using 3 μM of motor, 0.5 mM ATP, and 0–5 μM of MTs. Inorganic phosphate was measured using a colorimetric assay described in [12].

The MT gliding assay and cellular imaging were performed as described in [9]. Fixed cells were labeled with a tubulin antibody [13] and Hoechst 33342. Cell images are displayed as 2D maximum intensity projections following deconvolution, and the panels were assembled in Adobe Photoshop, Elements 15, San Jose, CA, USA).

### 2.3. Hairpin Constructs

The long hairpin constructs were assembled using PCR-generated fragments as outlined in Figure S1 [14]. The initial segments (Dd*kif8* (DDB_G0284471) [8], bp +49 to +714; and Dd*kif10*, bp +47 to +763) were amplified with primers containing 5′ *Not*1 and 3′ *Sal*1 restriction sites, and cloned in reverse orientation relative to the DdActin15 promoter in pLD1A15SN [15]. The second segments (Dd*kif8*, +343 to +714; and Dd*kif10*, +400 to +763) were amplified with primers containing 5′ *Not*1 and 3′ *Mlu*1 restriction sites and were cloned downstream but in a forward orientation adjacent to the initial segment. The constructs were sequenced to confirm orientation, junctions, and PCR fidelity. The expression of a single RNA transcript that folds into a hairpin, with a large unpaired loop (Dd*kif8*, 322 bases; Dd*kif10*, 353 bases) was predicted. The constructs were introduced into Dd*kif8* or Dd*kif10* null cells [7] by electroporation [16], and then clones were selected for growth in G418 (10 μg/mL).

## 3. Results

DdKif10 is a 1238aa (139 kDa) polypeptide with an amino-terminal motor domain, a central region with two prominent predicted coiled-coil motifs, and a carboxy terminal tail that likely contains cargo-binding regions [17,18]. The motor/neck domain clearly groups to the Kinesin-8 family, with 45% identity over the first 425 residues to the human Kif18A protein. There is no global homology in the remaining sequence to kinesins outside of the Dictyostelids; in part, this may be due to the frequency of amino acid repeats in *D. discoideum* coding regions that challenges simple BLAST comparisons (e.g., a stretch of 46 asparagine residues beginning at N_729_). However, important here is that even more local comparisons do not reveal significant similarity to either HsKif18A or ScKip3, particularly within regions of their tail known to contain a second, non-ATP sensitive MT binding domain (HsKif18A 777-898aa [19], ScKip3 447-805aa [20]).

**Figure 1:**
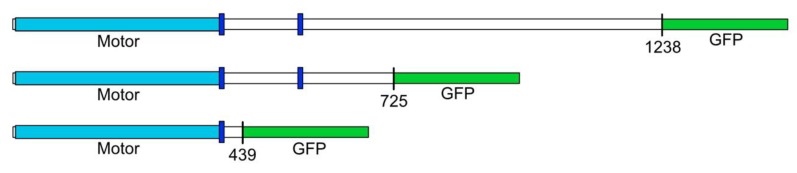
A schematic of the DdKif10 kinesin motor constructs. The position of the motor domain is indicated in light blue, and the dark blue boxes mark prominent coiled-coil motifs in the coding sequence. The top row represents the full-length, 1238aa construct. A detailed representation of the domain organization of DdKif10 can be found in reference [17]. Despite multiple attempts, we were only able to detect the expression of the middle-length construct in *D. discoideum* cells.

We developed three expression constructs to produce different-size tagged versions of DdKif10 in *D. discoideum* for analysis (Figure 1). Despite multiple previous successes using this strategy with other motors [9,21,22], we were unable to isolate *D. discoideum* clones that had detectable expression for either the full length or the motor-only domain constructs. A mid-length construct (725aa) containing a bit over half of the DdKif10 polypeptide did express as expected, and we were able to isolate this motor fragment for further analysis.

**Figure 2:**
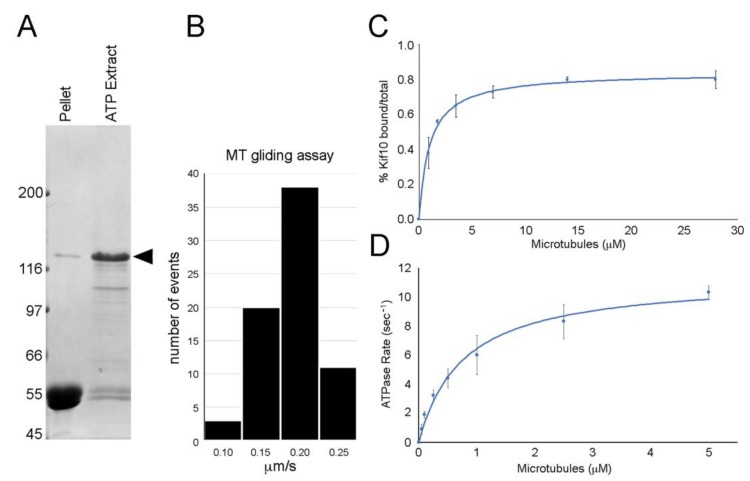
Motor mechanics. Panel (**A**) shows microtubule (MT) pellet and ATP extract lanes following the binding and release of the DdKif10_725_ polypeptide (Coomassie-stained gel, arrowhead denotes position of the Kif10 fusion protein). Panel (**B**) shows a histogram of MT gliding activity induced by the DdKif10_725_ fragment, with an average rate of 0.17 μm/s. Panel (**C**) shows the MT affinity of the motor fragment, plotting % pelleted vs. MT concentration. Panel (**D**) illustrates motor catalytic activity, plotting the ATPase rate vs. MT concentration. The data in both panels (**C**) and (**D**) represent averages from three independent measurements, and error bars indicate standard deviations.

The 110 kDa carboxy-terminal GFP fusion product of the mid-length construct (DdKif10_725_) readily bound to MTs in an ATP-sensitive fashion (Figure 2). In simple MT gliding assays, this motor induced MT motility at an average rate of 0.17 μm/s (± 0.036 SD, *n* = 71). MTs were seen to attach to the coverslip, glide smoothly over a distance, then readily detach and diffuse away. There was no evidence of MT bundling (with or without ATP) or MT depolymerization activity in similar mix and match assays.

We measured the affinity of the motor fragment to MTs, plotting % pelleted vs. MT concentration. The data were fitted to a modified Hill equation [23] and demonstrate a Kd [MT] of 0.99 ± 0.25 μM. The Hill coefficient is calculated to be 1.0, indicative of non-cooperative binding. We further measured the motor catalytic activity, plotting the ATPase rate vs. MT concentration. Data were fitted to the Michaelis-Menten equation, and illustrate a Km [MT] of 0.76 ± 0.25 μM and a Vmax of 11.3 (ATP/s). These results demonstrate an active MT-based motor.

**Figure 3:**
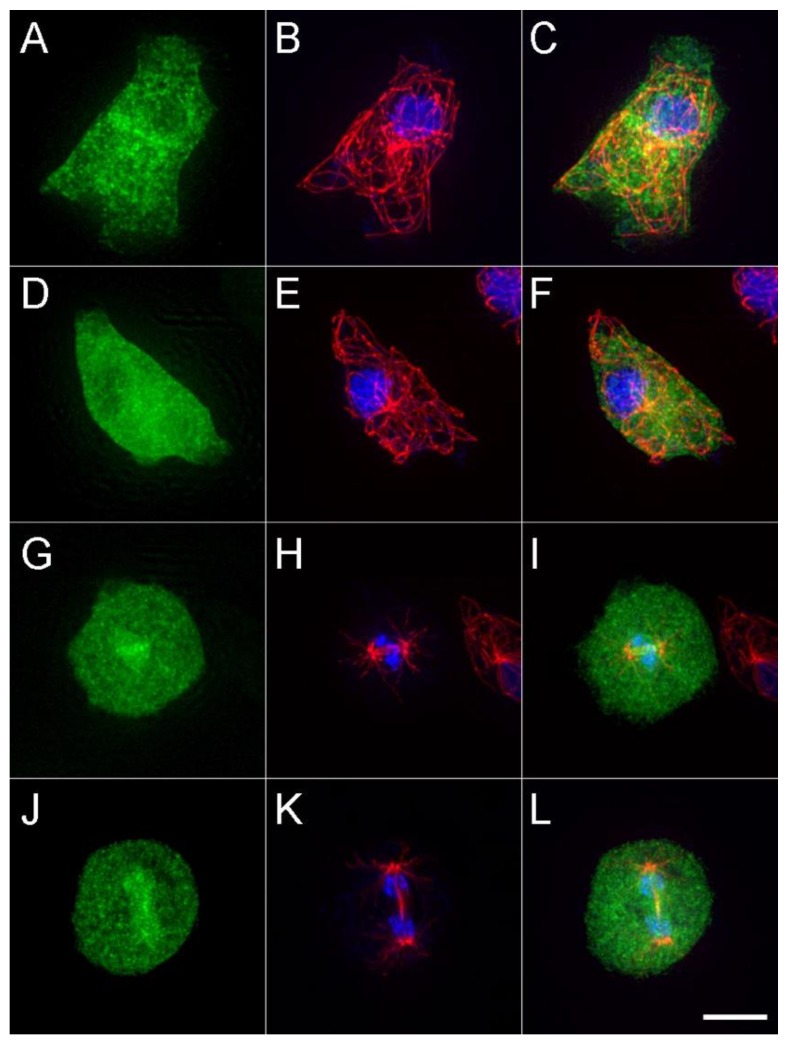
The cellular distribution of DdKif10_725_. The left column (green) shows the maximum intensity projections of the DdKif10 distribution in two interphase (**A**,**D**) and two mitotic cells (**G**,**J**). Antibody-labelled MTs (red) and Hoechst-stained nuclei (blue) are shown in the middle column (panels **B**,**E**,**H**,**K**), and the two frames are merged in the right column (panels **C**,**F**,**I**,**L**). Scale bar = 5 μm.

We examined the cellular distribution of the expressed motor construct (Figure 3). During interphase, there is a punctate pattern broadly distributed across the cytoplasm, with darkened areas that correspond to nuclei. There does not appear to be any enrichment along MTs, nor labeling at their plus ends. During mitosis, the motor accumulates within the nuclear compartment and, in some cells, appears to be enriched in the immediate area around the central spindle MTs. This result may indicate binding to the spindle MTs, or they may just be co-associated because of the structure of the nuclear envelope and spacing of the chromatin. Despite the abundance of the DdKif10_725_ in the cytoplasm, the MT arrays appear indistinguishable in length and number from those in WT cells.

**Figure 4:**
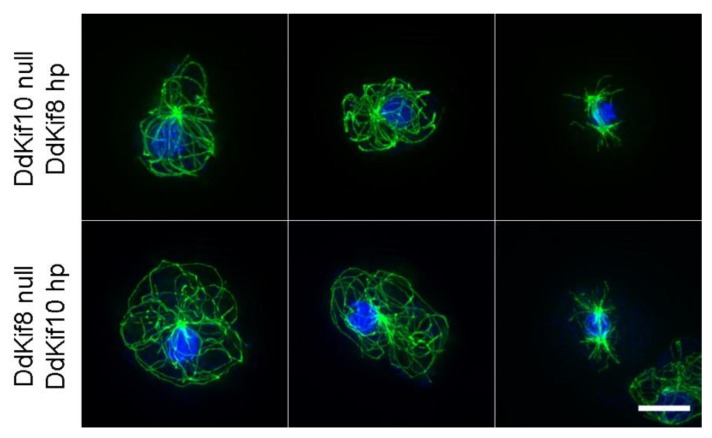
Complementary hairpin analysis. The top row shows MT arrangements (green) in three DdKif8 null cells (two interphase, one mitotic) in the presence of a constitutively expressed Dd*Kif10* hairpin construct. The bottom three rows show the same arrangement, but with the null/hairpin constructs reversed. In all cases, the MT arrays look no different than those seen in wild-type cells. Nuclei and chromosomes are shown in blue. Scale bar = 5 μm.

We also tested the idea that DdKif10 and DdKif8 cooperate in some capacity to organize the interphase MT array, providing MT pushing forces as a counterbalance to dynein-mediated pulling [24]. We expressed complementary RNAi hairpin constructs in Dd*kif* null cells (Dd*Kif8*hp in Dd*kif10* null cells, and vice versa). Similar RNAi strategies have proven effective in targeting the functional activities of the essential Dd*Kif6* gene [25] and multiple other proteins in this model organism [14,26,27]. However, the attempted knockdown of either DdKif8 or DdKif10 expression in complementary null cells produced no visible alteration to interphase or mitotic MT arrays (Figure 4).

## 4. Discussion

Kinesin-8 motors are generally considered slow, processive, and accumulate at the MT plus ends where they effect tip dynamics [2]. Our results indicate that the DdKif10 motor domain can move MTs in vitro at about the same rate as the human Kinesin-8 isoform (170 nm/s (Dd) vs. 128–299nm/s (HsKif18A [28])). This is slower than DmKlp67A (417 nm/s [29]) but faster than their fungal counterparts (7–39 nm/s (SpKlp5/6) [30,31]) and 10–60 nm/s (ScKip3) [32,33]). Our results do not address how processive this motor fragment might be, but its catalytic activity and solution MT affinity are consistent with those of other members of this family [20,28,30,31,32,34].

In some metazoans [35,36,37], Kinesin-8 motors appear targeted or sequestered into the nuclear compartment during interphase, and decorate spindle MTs during mitosis, often clustering at the MT plus ends. In fungi, the motor is cytoplasmic during interphase, where it strongly labels cytoplasmic MTs, and is then enriched in the nuclear compartment and onto spindle MTs during mitosis [3,5,6,32]. The DdKif10_725_ motor is similar, in part, to the latter scenario, broadly found in the cytoplasm and absent from the nuclear volume during interphase, but as cells enter mitosis, it appears to migrate into the semi-closed nuclear compartment and form punctate enrichments in the immediate spindle region. However, in contrast, our in vivo imaging indicates that the DdKif10_725_ motor does not accumulate on interphase or mitotic MTs. We recognize we are unable to express a full-length version with a proper tail domain, but MT decoration is also seen with a variety of expressed motor/neck versions similar in scale to DdKif10_725_, and in multiple organisms [5,28,38].

The punctate structures seen on or near *D. discoideum* spindles are not consistent among the mitotic cells we have observed, and thus we cannot say if they represent *D. discoideum* kinetochores or the spindle overlap region that Kinesin-8 targets in other organisms [2]. We cannot exclude a mitotic function for this motor in *D. discoideum*; however, gene knockouts or knockdowns of this isoform have no obvious effect on cell growth that would implicate a major role in cell division.

As a function of their affinity to MTs and particularly the plus ends, Kinesin-8 motors are generally known to effect MT dynamics, either through direct depolymerization or through effecting MT catastrophe [2]. In most cases, deletions of the motor result in visibly longer MTs in the cell whereas overexpression results in shortened or absent MTs. The *S. cerevisiae* motor (ScKip3) [32,33] and the human Kif18A [34,36] motor can actively depolymerize MTs, and in situ depolymerization effects are seen even with some truncated motor/neck combinations [32,37]. We do not see similar activities for the DdKif10_725_ motor, during interphase, mitosis, or in vitro assays. Within the motor domain, the loop 2 region of Kinesin-8 and -13 has been implicated in MT interaction and/or managing MT dynamics, and more recently in facilitating stable interactions with the plus ends of K-fiber MTs [39,40,41]. Despite strong overall sequence identity to HsKif18A in the motor/neck region, the short loop-2 sequence of the DdKif10 is largely unrelated (Figure S2). In particular, the DdKif10 sequence lacks the six positively charged lysine residues that were shown to be critical for HsKif18A accumulation at K-fiber plus ends [39]. This difference may help to explain the lack of accumulation along MTs, and perhaps a catalytic activity that perturbs MT structure. In addition, while *D. discoideum* MTs show plus end tip dynamics [24,42], overall, the interphase polymer is fairly stable and does not grow or shrink to any great extent. These dampened MT dynamics may also reflect the lack of DdKif10 MT binding and depolymerase activity in this organism.

Finally, our previous work suggests that DdKif10 collaborates with DdKif8 (Kinesin-4) to counterbalance dynein pulling activity during interphase in *D. discoideum*, acting in sum to support the radial MT array [7,18]. Laser cutting of the motile comet-like MT arrays induced by dominant-negative expression of dynein fragments indicates a pushing component that drives MT motility (MT plus-end-directed) [24], and we are unable to produce this distinctive MT behavior in either kinesin null cell background. These results lead to the idea that Dd Kinesin-4 and -8 push in some capacity, while dynein pulls on MTs. We tested a combined role, here, through targeted protein knockdowns in null cells, in an attempt to reduce or eliminate both DdKif10 and DdKif8, and could find no alteration of MT organization. We recognize that our current results do not quantitate reductions in DdKif protein levels, and thus we cannot rigorously exclude cooperative actions of these two motors. However, this strategy has proven effective in other applications [14,25,26,27], and thus we are confident that we would have seen some effect should there be an essential interaction between these two proteins. As a result, we cannot further explain how the MT array is pushed in the cytoplasm when dynein function is impacted.

## 5. Conclusions

Despite strong consistent phenotypes in multiple organisms, the function of the Kinesin-8 isoform in *Dictyostelium* remains an enigma. On the one hand, it is a bona fide MT-based motor with movement and catalytic properties, but it does not show localization or some of the functional activities shared with isoforms from other organisms that would readily illustrate a cellular role during interphase or mitosis. It seems unlikely that a motor would persist in a genome without an impactful function, and thus these results underscore the challenges in studying individual members of a diverse gene family.

## Supplementary Materials

The following are available online at https://www.mdpi.com/2218-273X/10/4/563/s1, Figure S1: RNAi hairpin schematics. Figure S2: Loop 2 comparison.
