# Supplementary material for: Bioenergetics of the Dictyostelium Kinesin-8 Motor Isoform
Source: Biomolecules. 2020 Apr 7;10(4):563. doi: 10.3390/biom10040563 (PMC7226124; doi:10.3390/biom10040563)
Supplement: Supplementary file 1 [file biomolecules-10-00563-s001.pdf]

biomolecules

Supplementary Materials: The following are available online at www.mdpi.com/xxx/s1, Figure S1: RNAi hairpin schematics. Figure S2: Loop 2 comparison.

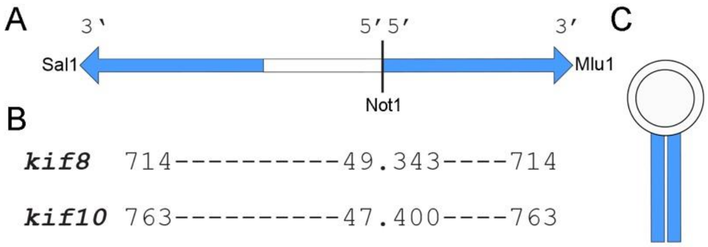

Figure S1. RNAi hairpin schematics. (A) The blue regions represent complementary sequences and their orientations for RNA base pairing, while the white region denotes the unpaired loop. (B) The numbers represent the DNA coding sequences used for hairpin construction. Panel C illustrates the final hairpin configuration following transcription.

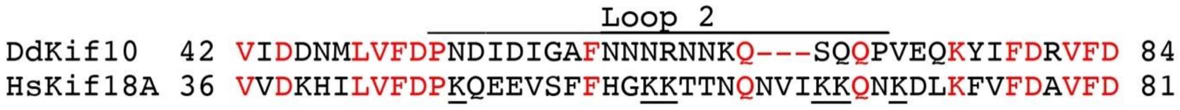

Figure S2. Loop 2 comparison. Sequence comparison in the loop-2 region between the *D. discoideum* and human Kinesin-8 isoforms. The red-colored letters highlight identical amino acid residues. The underlined Ks are identified as key lysine residues contributing to microtubule (MT)-tip binding in the human sequence [39].
